# Point-of-Care Ultrasound for Jugular Venous Pressure Assessment: Live and Online Learning Compared

**DOI:** 10.7759/cureus.1324

**Published:** 2017-06-08

**Authors:** Steve Socransky, Eddy Lang, Rhonda Bryce, Martin Betz

**Affiliations:** 1 Emergency Medicine, Northern Ontario School of Medicine; 2 Emergency Medicine, University of Calgary; 3 Clinical Research Support Unit, University of Saskatchewan; 4 Emergency Medicine, Sudbury Regional Hospital

**Keywords:** point-of-care diagnostics, emergency medicine, ultrasound, medical education, distance education

## Abstract

**Introduction:**

Point-of-care ultrasound (POCUS) is a novel technique for the assessment of jugular venous pressure. Distance education may allow for efficient dissemination of this technique. We compared online learning to a live course for teaching ultrasonography jugular venous pressure (u-JVP) to determine if these teaching methods yielded different levels of comfort with and use of u-JVP.

**Methods:**

This was an interventional trial of Canadian emergency physicians who had taken a basic POCUS course. The participants were in one of three Groups: online learning (Group OL), live teaching (Group LT), control (Group C). Group LT participants also took an advanced course prior to the study that included instruction in u-JVP. The participants who took the basic course were randomized to Group OL or Group C. Group OL was subject to the intervention, online learning. Group C only received an article citation regarding u-JVP. Questionnaires were completed before and after the intervention. The primary outcome was physician self-reported use and comfort with the technique of u-JVP after online learning compared to live teaching.

**Results:**

Of the 287 advanced course participants, 42 completed the questionnaires (Group LT). Of the 3303 basic course participants, 47 who were assigned to Group OL completed the questionnaires and 47 from Group C completed the questionnaires. Use of u-JVP increased significantly in Group OL (from 15% to 55%) and Group C (from 21% to 47%) with the intervention. The comfort with use did not differ between Group LT and Group OL (p=0.14). The frequency of use remained higher in Group LT than Group OL (p=0.07).

**Conclusion:**

Online learning increases the use and comfort with performing u-JVP for emergency physicians with prior POCUS experience. Although the comfort with use of u-JVP was similar in Groups LT and OL, online learning appears to yield levels of use that are less than those of a live course.

## Introduction

The evaluation of jugular venous pressure (JVP) is a standard part of the physical examination in patients with dyspnea, particularly when acute congestive heart failure is suspected [1]. Unfortunately, assessment of JVP by visualization of jugular venous pulsations is inaccurate [2-5]. Factors such as short or obese necks make this evaluation difficult or impossible [6]. As a result, physicians may not incorporate JVP into diagnostic decision-making.

The assessment of JVP using bedside ultrasound (u-JVP) represents an alternative to JVP determination by physical exam (e-JVP). The internal jugular vein is readily identified by ultrasound. The technique of using point-of-care ultrasound (POCUS) to determine JVP was first described by Lipton [7]. One small study suggested that an elevated u-JVP may be more accurate than a chest radiograph in diagnosing heart failure [8]. The normal parameters for u-JVP have been defined [9].

Some physicians with experience in POCUS have reported that u-JVP is a simple technique to learn [7]. Despite its simplicity, the availability of continuing medical education in this technique is limited by both the small number of courses that teach it and the limited number of physicians who can attend such courses. Distance education presents an alternative method for learning u-JVP, which may permit its more efficient dissemination. Although distance education for POCUS holds promise [10-14], it is unclear whether one method is preferable over the other for the acquisition of the skill set needed to perform a simple, non-invasive, and low-risk POCUS technique such as u-JVP.

We developed a web-based teaching module with cognitive and motor components for teaching u-JVP. This study compared online learning to a live course for teaching how to perform u-JVP. The objective was to determine if effectiveness differs between a live course and online learning in teaching the u-JVP technique to physicians.

## Materials and methods

### Study design

This study was an interventional trial of web-based learning that took place from May to October 2011. The study participants were Canadian emergency physicians (EPs) who had taken a basic POCUS course. Study approval was provided by the Research Ethics Committee of Health Sciences North.

### Study setting and population

Canadian EPs who had taken a basic POCUS course (The EDE Course, www.edecourse.com) were the target population of the study. Over 5000 physicians had taken the basic course at the time of the study. The basic course does not include training in u-JVP. Physicians who had also taken an advanced POCUS course that does include training in u-JVP (The EDE 2 Course, www.ede2course.com) represented one of the comparator groups. Over 200 physicians had taken the advanced course at the time of the study. An article published in the Canadian Journal of Emergency Medicine (CJEM) in 2010 was used as a u-JVP learning tool for a third comparator group [9]. Upon completion of a basic POCUS course, physicians have the option of obtaining independent practitioner (IP) status with the Canadian Emergency Ultrasound Society (CEUS) by performing 200 training scans under direct supervision.

### Study protocol

**Figure 1:**
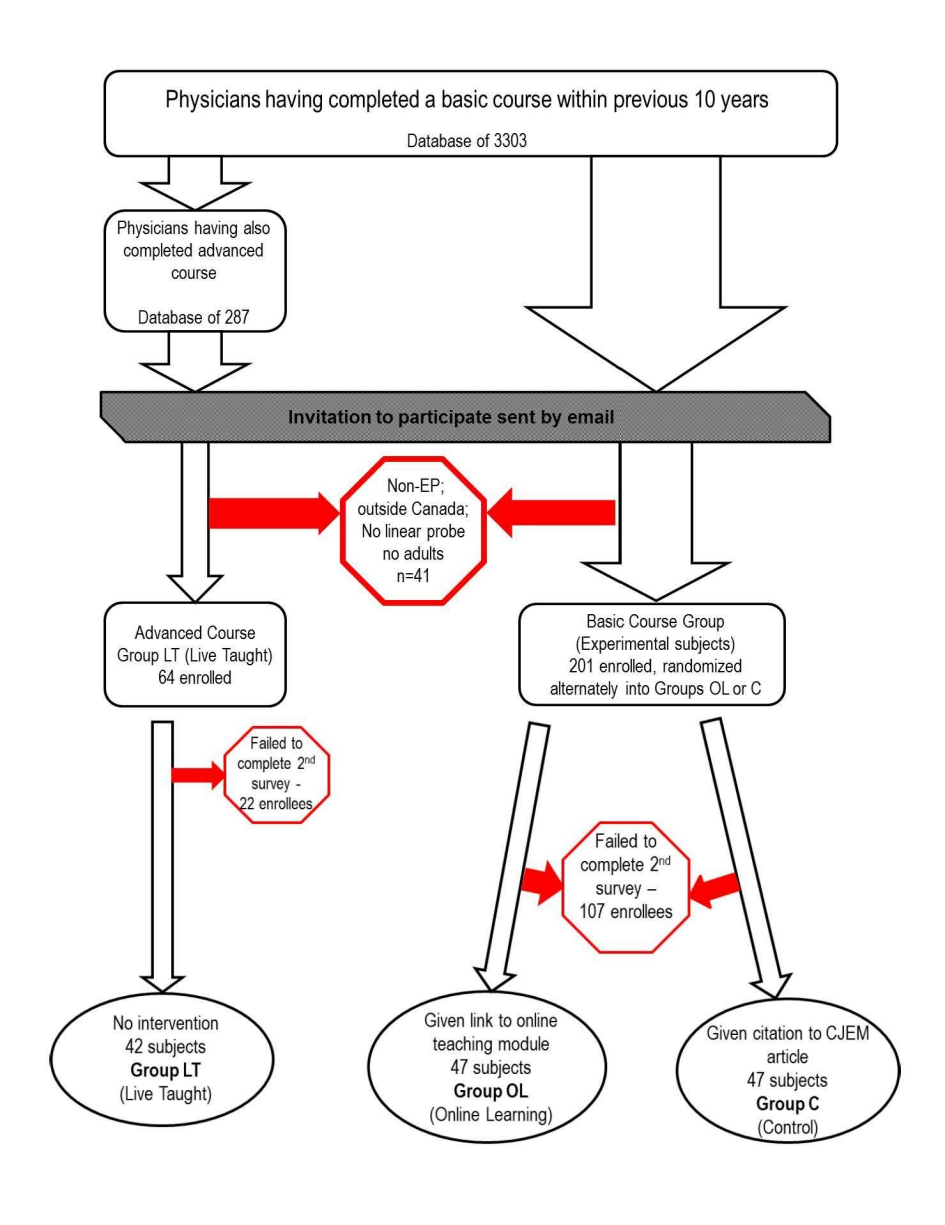
Graphic outline of study protocol EP: emergency physician, Group OL: online learning group, Group LT: live teaching group, Group C: control group.

There were three study groups: an intervention group (Group OL – Online Learning) and two comparison groups (Group LT – Live Teaching and Group C – Control). Figure 1 depicts how participants were selected for each of the groups. Subjects were recruited to Group LT from a database of email addresses for the 287 physicians who had been participants of the advanced emergency ultrasound course. Subjects were recruited to Group OL and C from a database with the available email addresses of 3303 physicians who had been participants of the basic course since 2001.

Invitations to participate in the study were sent by email to course participants. After providing informed consent, respondents were directed to an initial online questionnaire, which screened for inclusion and exclusion criteria. Respondents were included in the study if they were EPs working in Canada, saw adult patients, and had access to an ultrasound machine with a linear array probe. Physicians were excluded if they were not EPs, did not work in Canada, saw only children, or did not have access to a linear array probe.

Participants from the two different databases who provided consent and met inclusion criteria were then directed to different initial questionnaires. The questionnaires queried demographics, emergency medicine and POCUS training and experience, practice setting, and prior use of e-JVP. Groups OL and C were also questioned regarding their knowledge and prior use of u-JVP. Group LT was already exposed to u-JVP teaching material at a live course held between 2009 and 2011. Subsequent to this initial questionnaire, Group LT participants had no intervention and received no further communication for six months.

Upon completion of the first questionnaire, respondents from the basic course database were randomized alternately from an alphabetical list into Group OL or Group C. Group OL, subject to the intervention, received a link to an online teaching module which included a downloadable text in PDF format [15] and a video recording of a live lecture in PowerPoint format which could be viewed an unlimited number of times. There was no opportunity to interact with instructors online. Only basic technical problems related to computer access to the content were addressed. In contrast, Group LT was already exposed to u-JVP training at the live course, which included the same downloadable text delivered prior to the course, a lecture, and a workshop, as well as an opportunity to interact with instructors.

Participants assigned to Group C were simply provided with a citation to an article [9] that described the technique of measuring u-JVP. They were not provided with the actual article. Membership with the Canadian Association of Emergency Physicians or personal/institutional journal subscription was required to have free access to the article. Groups OL and LT also received the citation as part of the references section of the downloadable text.

Six months following the completion of the first questionnaire and subsequent to the intervention, all participants were asked to complete a second questionnaire, which was different for each of the three groups. All questionnaires asked about the respondents’ comfort level, use, perceived utility of u-JVP, and if they showed another physician how to perform it. They were also asked if they read the CJEM article about u-JVP. Group OL was also asked if they had any difficulty with access to the online materials and if they reviewed them.

### Measured outcomes

The primary outcome was physicians’ self-reported use and comfort with the technique of u-JVP after online learning (Group OL) compared to a live course (Group LT). Online learning was also compared with passive uptake of the technique by physicians who may have been exposed to a journal article describing the performance of u-JVP (Group C). A secondary outcome was the rate of successful access to and completion of the online learning module.

### Data analysis

Online questionnaires were administered using Survey Monkey (www.surveymonkey.com) (SurveyMonkey, CA, USA). Upon survey completion, the responses were downloaded and collated on a standardized Microsoft Excel spreadsheet (Microsoft Corporation, Redmond, WA). Data were analyzed only for participants who completed both questionnaires, although each individual’s pre- and post-intervention responses could not be matched and were thus treated independently. Using SAS software (version 9.4, SAS Institute Inc., Cary, NC, USA), chi-square testing was utilized to determine statistical differences in responses. Fisher’s exact test was used if more than 20% of expected cell counts were less than five. If a statistical difference was detected (p < 0.05), further pairwise testing was performed (LT vs OL, LT vs C, OL vs C) to determine which differed significantly at a Bonferroni-corrected alpha value.

A sample size calculation was performed to determine that a sample of 97 patients in each group was needed to provide the study with 80% power to detect a 20% difference in the use of u-JVP between Groups LT and OL on two-sided testing with alpha set at 0.05. The 20% difference was estimated by the authors as the difference required for one teaching method to be chosen over another. Four hundred responses to the initial invitations were predicted. After consent and completion of the first questionnaire, it was estimated that the dropout rate would be approximately 20%.

## Results

Of the 287 advanced course participants, 64 (22%) provided informed consent and completed the first questionnaire. All met the inclusion criteria. Forty-two of 64 (15%) completed the second questionnaire and were included in Group LT.

Of the 3303 basic course participants, 289 provided informed consent (9%). However, 88 were excluded because they took the advanced course (n=47), only saw children (n=4), did not have a linear array probe (n=27), or worked outside of Canada (n=10). Of the 201 remaining responders (6%), 100 were assigned to Group OL and 101 were assigned to Group C. A total of 94 physicians (3%) completed the second questionnaire: 47 from Group OL and 47 from Group C.

Demographic and practice characteristics across the groups are shown in Table 1. Each group had a similar age distribution, but there were significant differences in emergency department (ED) work experience between the groups. Fifty-one per cent of Group OL reported working less than six years in the ED, a larger proportion than in Groups LT or C (31% and 26%), while Group C subjects frequently indicated more than 10 years. A larger proportion of physicians in Group LT (71%) reported using POCUS for more than three years, as compared to the subjects in Groups OL and C. Similarly, a larger proportion of Group LT participants reported having IP certification (90%) compared to either Groups OL or C (34%, 40%). The proportion of participants having emergency medicine credentials was similar in each group. All groups worked in similar EDs (rural vs urban, annual census, geographic location), but EPs in Group LT worked more shifts per month in busier EDs. Teaching responsibilities were similar in the three groups. Group LT was more likely to have used e-JVP in the last year.

**Table 1:** Characteristics of study participants based on group Group LT: live teaching group, Group OL: online learning group, Group C: control group, POCUS: point-of-care ultrasound, EM: emergency medicine, e-JVP: jugular venous pressure measurement by physical exam.

Characteristic		Group LT [n 42]	Group OL [n 47]	Group C [n 47]
		Number (%)	Number (%)	Number (%)
Age (y)	< 31	4 (10)	11 (23)	7 (15)
	31 – 40	22 (52)	17 (36)	14 (30)
	> 40	16 (38)	19 (40)	26 (55)
ED experience (y)	< 6	13 (31)	24 (51)	12 (26)
	6 – 10	13 (31)	6 (13)	7 (15)
	>10	16 (38)	17 (36)	28 (59)
POCUS experience (y)	<1	3 (7)	14 (30)	11 (23)
	1 – 3	9 (21)	28 (60)	20 (43)
	> 3	30 (71)	5 (11)	16 (34)
POCUS certification* (CEUS)		38 (90)	16 (34)	19 (40)
Credentials	Any EM certification	31 (74)	27 (57)	30 (64)
	No EM certification	7 (17)	17 (36)	13 (28)
	In training (residency)	4 (10)	3 (6)	4 (9)
ED work place	Urban	37 (88)	34 (72)	35 (74)
	Small town, rural	5 (12)	13 (28)	12 (26)
ED annual census	<30,000	5 (12)	8 (17)	9 (19)
	30-50,000	9 (21)	21 (45)	16 (34)
	>50,000	28 (67)	18 (38)	22 (47)
Region of Canada	West‡	12 (29)	13 (28)	14 (30)
	Ontario	20 (48)	17 (36)	19 (40)
	Quebec	7 (17)	10 (21)	11 (23)
	Atlantic Canada^§^	3 (7)	7 (15)	3 (6)
ED clinical workload (shifts/month)	< 9	4 (10)	16 (34)	11 (23)
	9 – 12	9 (21)	14 (30)	18 (38)
	13 – 16	23 (55)	12 (25)	16 (34)
	> 16	6 (14)	5 (11)	2 (4)
Teaching responsibilities	Teaching centre	24 (57)	16 (34)	18 (38)
	Trainees frequently	17 (40)	24 (51)	24 (51)
	Trainees rarely/never	1 (2)	7 (15)	5 (10)
Use of e-JVP in last year	At least once	36 (86)	7 (15)	10 (21)
	Never	6 (14)	40 (85)	37 (79)
Confidence in e-JVP results	Very confident	4 (10)	4 (9)	6 (13)
	Somewhat confident	19 (45)	28 (60)	23 (49)
	Not confident	19 (45)	15 (32)	18 (38)
* ED ultrasound certification by Canadian Emergency Ultrasound Society (CEUS)				
† Canadian College of Family Practice-Emergency Medicine, Fellow of Royal College of Physicians, Fellow of American College of Emergency Medicine, American College of Emergency Physicians				
‡ British Columbia, Alberta, Saskatchewan, Manitoba, Yukon				
§ Newfoundland & Labrador, Prince Edward Island, Nova Scotia, New Brunswick				

The first questionnaire revealed that seven (15%) of Group OL and 10 (21%) of Group C participants had been informally taught the technique of u-JVP by someone who had received formal training. At the time of the second questionnaire, none of the Group OL participants and four (8%) of Group C subjects had received live teaching of this technique.

Comfort with the technique of u-JVP for all groups at the time of the second questionnaire is shown in Table 2. Eighty-six per cent of Group LT believed that u-JVP was very easy or not difficult to perform. All but four (96%) participants from the web-based learning group felt the same. Forty-nine percent of the control group felt the technique to be easy or not difficult while another 36% had not tried it or did not answer the question. There was a statistically significant difference between the groups in self-reported ease of use or failure to try. Group LT was considerably more likely than Groups OL and C to teach u-JVP to others.

**Table 2:** Comparison of group questionnaire responses post-intervention JVP: jugular venous pressure, u-JVP: measurement of JVP using ultrasound, Group LT: live teaching group, Group OL: online learning group, Group C: control group.

Measuring JVP by ultrasound (u-JVP) as Reported by Participants	Group LT [n 42] Number (%)	Group OL [n 47] Number (%)	Group C [n 47] Number (%)	p-values
				(Chi-square testing)
				Ease of performing u-JVP				Overall: p<0.0001
Very easy or not too difficult	36 (86)^†^	45 (96)^‡^	23 (49)^†‡^	LT vs OL p=0.14*
Somewhat or very difficult	6 (14)	2 (4)	7 (15)	LT vs C p<0.0001
Not tried/no answer	0 (0)^†^	0 (0)^‡^	17 (36)^†‡^	OL vs C
Frequency of use of u-JVP				Overall: p=0.005
At least monthly	19 (46)^†^	7 (15)^†^	13 (28)	LT vs OL
Less than monthly	12 (29)	19 (40)	9 (19)	LT vs C
Never used	11 (26)	21 (45)	25 (53)	OL vs C
Trained others in use of u-JVP				Overall: p=0.0002
Yes	21 (50)^†^	5 (11)^†^	12 (26)	LT vs OL p<0.0001
No	21 (50)^†^	42 (89)^†^	35 (74)	LT vs C p=0.02
				OL vs C p=0.06
Usefulness of ultrasound to measure JVP				Overall: p=0.98
Very useful	8 (19)	9 (19)	8 (17)	
Possibly useful	28 (67)	32 (68)	31 (66)	
Probably not useful	6 (14)	6 (13)	8 (17)	
Paired symbols within each row († and ‡ respectively) indicate categories found to differ between groups.				
*Fisher’s exact test				

Prior to the intervention, self-reported use of the technique of u-JVP in Groups OL and C was low (15% and 21%, respectively), reflecting the low rate of prior training in the technique (Table 3). Six months later, self-reports of using the technique increased significantly to 55% (p<0.0001) in Group OL and 47% (p=0.009) in Group C. Although there was an insignificant drop from 86% after six months (p = 0.28), the use of u-JVP in Group LT remained higher (75%) than in Groups OL or C. When asked whether u-JVP was, or could be, clinically useful, a similar proportion of participants in each group answered in the affirmative. Of the 136 participants, the number who felt u-JVP was probably not useful rose from two initially to 20 at the time of the second questionnaire.

**Table 3:** Assessment of change in ultrasound use and perceived usefulness within groups pre and post intervention Group LT: live teaching group, Group OL: online learning group, Group C: control group, JVP: jugular venous pressure, u-JVP: measurement of JVP using ultrasound. *Pre-intervention, the OL Group was missing responses for 20 subjects (43%) and the C Group for 15 subjects (32%). All subjects responded to this question post intervention. †Fisher’s exact test.

	Group LT [n 42]		p-value	Group OL [n 47]		p-value	Group C [n 47]		p-value
	Number (%)			Number (%)			Number (%)		
	Pre Intervention	Post Intervention		Pre Intervention	Post Intervention		Pre Intervention	Post Intervention	
Uses ultrasound to measure JVP (u-JVP)			0.28			<0.0001			0.009
Yes	36 (86)	31 (75)		7 (15)	25 (55)		10 (21)	22 (47)	
No	6 (14)	11 (26)		40 (85)	21 (45		37 (79)	25 (53)	
Usefulness of u-JVP*			0.17†			0.27†			0.01†
Very useful	9 (21)	8 (19)		9 (33)	9 (19)		12 (38)	8 (17)	
Possibly useful	31 (76)	28 (67)		17 (63)	32 (68)		20 (63)	31 (66)	
Probably not useful	1 (2)	6 (14)		1 (1)	6 (13)		0 (0)	8 (17)	

The u-JVP article [9] was read by a minority of all participants in each group at the beginning of the study (Table 4). At the time of the second questionnaire, the proportion of subjects who reported at least reading the abstract increased in all groups (Table 4). Anecdotally, several Group C participants commented that they would have liked to receive the article, rather than just the citation.

**Table 4:** Awareness and utilization by group of journal article on measuring JVP by ultrasound (u-JVP) Group LT: live teaching group, Group OL: online learning group, Group C: control group, u-JVP: measurement of jugular venous pressure using ultrasound.

	Group LT [n 42]		Group OL [n 47]		Group C [n 47]	
	Number (%)		Number (%)		Number (%)	
u-JVP article (9)	Pre Intervention	Post Intervention	Pre Intervention	Post Intervention	Pre Intervention	Post Intervention
Read all or part	16 (38)	25 (60)	5 (11)	18 (38)	8 (17)	39 (83)
Did not read	4 (10)	17 (40)	7 (15)	29 (62)	7 (15)	8 (18)

Among the 47 participants in Group OL, 22 (47%) read all (or most) of the text and watched all (or most of) the video lecture, four (9%) read the text but did not watch the video, three (6%) watched the video but did not read the text, and 17 (36%) did neither. Difficulties were experienced by five participants (11%) in trying to download the text and four participants (9%) in trying to access the video.

## Discussion

This study compared three ways of learning a new POCUS skill: online learning, a live course, and article review. The two comparison groups (LT and C) represented more traditional methods of skill acquisition. The results suggest that online learning increases the use and comfort level of EPs learning a simple POCUS technique. However, online learning did not increase its use or the likelihood of teaching others to the same level as live teaching.

With the intervention, the use of u-JVP increased significantly in Group OL. With the sole intervention of being provided with the article citation, the use of u-JVP also increased significantly in Group C. However, its use in these groups was still significantly less than in Group LT. Of note, subjects in Groups LT and OL expressed similar ease with the procedure, regardless of instructional method. Larger proportions of subjects in Groups LT and OL found u-JVP easier to perform than Group C, although this discrepancy may be due to the one-third of subjects in Group C who did not provide an opinion. Surprisingly, the perceived usefulness of u-JVP decreased in all groups, reaching significance in Group C; like ease of use, these differences may be due in part to more subjects choosing to provide their opinion regarding usefulness after the intervention. Despite the intervention, Group LT participants were still significantly more likely to teach others in the technique than the other groups. Despite these differences between the groups, there was no difference between the groups in perceived usefulness of u-JVP post-intervention.

There are several characteristics that make this study novel compared to past studies of distance education [10, 12, 16]. First, the subjects of this study were mainly practicing physicians rather than resident trainees. Second, the subjects were unknown to the teachers and were recruited from across the country. Third, there was a third comparison group, a true control group, who did not receive any specialized instruction. Fourth, the duration of the study was six months, which is longer than most similar studies, although still not optimal [17].

The results of two other studies of web-based learning of POCUS skills have relevance to our study. A study by Platz [11] showed that there was no difference in knowledge acquisition in POCUS for trauma when delivered online vs a traditional classroom setting. However, this study had no workshop component. A study by Chenkin [10] similarly showed that web-based classroom approaches yielded the same knowledge and skill gains for POCUS-guided vascular access. The Chenkin study did have a workshop component that was the same for both groups, but the workshop was self-directed, with equipment available but without instructors present. Despite the baseline differences between Groups LT and OL, the presence of a workshop for Group LT may explain the differences in results between these groups. Another factor may be the on-demand nature of the online module, a method of learning shown to be inferior to scheduled web-based teaching sessions, at least for knowledge acquisition by community-based physicians [18].

The persistently higher use of u-JVP and greater likelihood of teaching others in Group LT may reflect a difference in training, specifically having had a practical session at the live course, not available to the other groups. It also may reflect longer experience with u-JVP. The greater ease of use in Group OL compared to Group C may be related to a more thorough description of the technique in the online learning resources compared to the article.

This study has a number of limitations. Similar to many studies of web-based learning, this study did not strictly adhere to all of the proposed recommendations for evaluation of effectiveness of continuing medical education [17]. For example, evaluation of outcomes was limited to assessment of participant attitudes. It did not include measure changes in physician practices or patient outcomes. Also, post-intervention follow-up occurred at six months rather than at one year. The online learning materials were only of intermediate sophistication.

All participants in Group LT watched the lecture and participated in the workshop. It is unknown if all Group LT participants read the text. Only 47% of Group OL participants read the text and watched the lecture. Over one-third did neither. The article on u-JVP [9] was read by 60% of Group LT participants, 38% of Group OL participants, and 83% of Group C participants. Motivation may be a reason why less than half of Group OL participants used all the learning materials. Group LT participants registered and paid for the live course and received CME credits. Group OL participants had less POCUS experience and were less likely to work full-time. Groups OL and C made less frequent use of e-JVP in their practices than Group LT.

Of the 287 participants of the advanced course, only 64 were enrolled in Group LT. Of the 3303 participants of the basic POCUS course, only 201 were enrolled in Groups OL and C. The post-intervention survey was completed by only two-thirds of physicians in Group LT and less than half of physicians assigned to Groups OL or C, each experiencing a similar dropout rate. It is unknown how this may have affected the results. An insufficient number of respondents led to fewer enrollments than was required based on the power calculation. Nonsignificant differences between groups may or may not have become significant with greater enrollment or if paired analysis of individual subjects’ pre- and post-intervention responses could have been undertaken.

## Conclusions

Online learning increases the use and comfort of performing the simple technique of measuring JVP using ultrasound for emergency physicians with prior POCUS experience. However, it appears to yield levels of use that are less than those of a live course that includes a practical workshop. Future research should focus on online learning of increased sophistication, more complex POCUS techniques, and different formats for practical workshops.
